# Characterising the dynamics of cerebral metabolic dysfunction following traumatic brain injury: A microdialysis study in 619 patients

**DOI:** 10.1371/journal.pone.0260291

**Published:** 2021-12-16

**Authors:** Mathew R. Guilfoyle, Adel Helmy, Joseph Donnelly, Matthew G. Stovell, Ivan Timofeev, John D. Pickard, Marek Czosnyka, Peter Smielewski, David K. Menon, Keri L. H. Carpenter, Peter J. Hutchinson

**Affiliations:** 1 Division of Neurosurgery, Department of Clinical Neurosciences, University of Cambridge, Cambridge, United Kingdom; 2 Division of Anaesthesia, Department of Medicine, University of Cambridge, Cambridge, United Kingdom; Uniformed Services University, UNITED STATES

## Abstract

Traumatic brain injury (TBI) is a major cause of death and disability, particularly amongst young people. Current intensive care management of TBI patients is targeted at maintaining normal brain physiology and preventing secondary injury. Microdialysis is an invasive monitor that permits real-time assessment of derangements in cerebral metabolism and responses to treatment. We examined the prognostic value of microdialysis parameters, and the inter-relationships with other neuromonitoring modalities to identify interventions that improve metabolism. This was an analysis of prospective data in 619 adult TBI patients requiring intensive care treatment and invasive neuromonitoring at a tertiary UK neurosciences unit. Patients had continuous measurement of intracranial pressure (ICP), arterial blood pressure (ABP), brain tissue oxygenation (PbtO_2_), and cerebral metabolism and were managed according to a standardized therapeutic protocol. Microdialysate was assayed hourly for metabolites including glucose, pyruvate, and lactate. Cerebral perfusion pressure (CPP) and cerebral autoregulation (PRx) were derived from the ICP and ABP. Outcome was assessed with the Glasgow Outcome Score (GOS) at 6 months. Relationships between monitoring variables was examined with generalized additive mixed models (GAMM). Lactate/Pyruvate Ratio (LPR) over the first 3 to 7 days following injury was elevated amongst patients with poor outcome and was an independent predictor of ordinal GOS (p<0.05). Significant non-linear associations were observed between LPR and cerebral glucose, CPP, and PRx (p<0.001 to p<0.05). GAMM models suggested improved cerebral metabolism (i.e. reduced LPR with CPP >70mmHg, PRx <0.1, PbtO_2_ >18mmHg, and brain glucose >1mM. Deranged cerebral metabolism is an important determinant of patient outcome following TBI. Variations in cerebral perfusion, oxygenation and glucose supply are associated with changes in cerebral LPR and suggest therapeutic interventions to improve cerebral metabolism. Future prospective studies are required to determine the efficacy of these strategies.

## Introduction

Traumatic Brain Injury (TBI) remains the leading cause of death and disability amongst young people (i.e. age <40 years), and the annual incidence has continued to increase over the last decade [1]. Death and neurological function after TBI are determined by a combination of the severity of the primary insult (i.e. the degree of immediate irreversible axonal disruption and cell loss due to mechanical forces at the time of impact), and the ensuing secondary processes exacerbating the extent of tissue injury over subsequent hours and days [2]. These latter processes are a potentially modifiable influence on patients’ outcome and are the focus of current and experimental interventions in neurointensive care.

Cerebral metabolic dysfunction has been described in acute brain injury of various aetiology, including TBI, ischaemic stroke, and intracerebral haemorrhage, and can potentiate secondary injury cascades such as induction of pro-inflammatory cytokines and microvascular breakdown causing blood brain barrier (BBB) permeability [3, 4]. Evidence from cell culture and tissue models, and from animal and human studies, has suggested several distinct states of metabolic dysfunction, broadly classified as cellular substrate deficiency (a consequence of tissue ischaemia or local diffusion barriers), aerobic hyperglycolysis, or a failure of oxidative phosphorylation that has been dubbed ‘mitochondrial dysfunction’ [5]. Moreover, in an experimental in-vivo study in piglets, mitochondrial dysfunction was manifested by changes measured by cerebral microdialysis—elevations in lactate concentration and lactate/pyruvate ratio, and decrease in glucose, accompanied by normal pyruvate, with normal PbtO_2_ [6]. This is distinct from cerebral ischemia that shows decreases in PbtO_2_ and pyruvate [6]. Distinguishing between these dynamic states within individual TBI patients *in vivo* is key to tailoring interventions that effectively ameliorate the extent of secondary injury.

Microdialysis is an invasive neuromonitoring technique that permits continuous real-time sampling of brain extracellular fluid and assessment of cerebral metabolism in the intensive care unit [7]. Metabolites assayed on bedside analysers include glucose, lactate, pyruvate, glutamate, and glycerol. There is a body of observational evidence to support absolute values, ratios, and temporal trends of specific metabolites as indicators of cerebral metabolic dysfunction. The most extensively studied parameter is the lactate/pyruvate ratio (LPR), generally accepted to be an index of cellular redox state and the balance between oxidative and anaerobic metabolism, and patients with average or prolonged LPR above thresholds of 25 or 40 are more likely to die or have unfavourable outcome following TBI [7]. Low cerebral glucose (approximate threshold of 0.8-1mM) has been associated with worse outcome both in observational series and interventional studies comparing alternative glycaemic control regimens, and there is also evidence that very high brain glucose is potentially deleterious [7, 8].

Characterising the relationships between elevated LPR and modifiable physiological parameters, aimed at defining therapeutic strategies to improve cerebral metabolism, is a priority for TBI research. However, the functional form of interactions between the key parameters in question are likely to be non-linear and analysis techniques need to be robust to the strong temporal autocorrelation in physiological data, if meaningful conclusions are to be drawn.

In this study we utilised microdialysis data from a large cohort of TBI patients with three principal objectives. Firstly, to quantify the independent effect of LPR and cerebral glucose on neurological outcome; secondly, to characterise the temporal course of these parameters following TBI, and thirdly, to assess the functional relationships between energy metabolites and other monitoring variables to inform an outline clinical protocol for managing traumatic metabolic dysfunction.

## Methods

### Patients

Data collection and analysis was approved by Local Ethical Committee and by the hospital Research and Development Department as part of our ongoing head injury program, and for all patients consent to the use of data was obtained from the next of kin or other legal representative.

This observational study includes 619 adult (>16 years) patients with TBI admitted for management on the neurocritical care unit (NCCU) at Addenbrooke’s Hospital, Cambridge, UK from 1997 to 2016. Data analysis from the first 223 patients has been published previously [8].

All patients required intubation and mechanical ventilation and were managed according to a standardised tiered therapy protocol. In brief, patients were sedated, paralysed, and mechanically ventilated to a target PaCO_2_ of 4.5–5 kPa. Vasopressors are used to maintain a cerebral perfusion pressure (CPP) of 55-65mmHg. In response to elevated intracranial pressure there was a staged application of osmotherapy, external ventricular drainage, hypothermia, barbiturate coma, and ultimately decompressive craniectomy. Six month neurological outcome was assessed with the Glasgow Outcome Score, which was dichotomised into favourable (GOS 4–5) and unfavourable outcome (GOS 1–3).

### Monitoring

Routine invasive neuromonitoring comprised of a triple lumen cranial access device (Technicam, Newton Abbott, UK) placed by default in the right frontal region unless contraindicated. An intracranial pressure (ICP) monitor (Codman, Raynham, MA, USA), a brain tissue oxygen (PbtO_2_) probe (Licox, Integra Neurosciences, Andover, UK), and a microdialysis catheter (CMA71, CMA/M Dialysis AB, Stockholm, Sweden; 100kDa molecular weight cut-off, or CMA70, 20kDa cutoff) were introduced via the access device and positioned with their tips in the white matter.

Microdialysis catheters were perfused with standard crystalloid perfusate (CNS perfusion fluid, CMA/M Dialysis AB, Stockholm, Sweden) at 0.3*μ*l/min. Microdialysate collection vials were changed hourly and assayed on bedside analysers (CMA 600, ISCUS, or ISCUSflex; CMA/ M Dialysis AB, Stockholm, Sweden) for concentrations of lactate, pyruvate, glucose, glutamate, and glycerol.

Arterial blood pressure (ABP) was monitored continuously via a peripheral intra-arterial catheter. ICP, ABP, and PbtO_2_ were digitised and recorded at 50-200Hz using ICM+ software (ICM+, Cambridge Enterprise/University of Cambridge, UK). ICM+ has the facility for online calculation of secondary indices including the PRx metric of cerebral autoregulation. This is quantified as the moving Pearson correlation coefficient between thirty successive mean values of ICP and ABP calculated over 10 second windows [9]. A positive PRx value suggests ICP changing in concert with ABP, indicative of poor (passive) autoregulation; PRx of approximately zero or negative indicates intact autoregulation [9].

### Data processing

The raw data files from the microdialysis analysers were parsed and imported using custom R scripts. Data were excluded if the analyte concentration was below the assay’s lower limit of detection (LLD) and/or there was insufficient microdialysate in a vial. LLDs of the analysers specified in the manufacturer’s manual were: glucose 0.1 millimol/L, lactate 0.1 millimol/L, pyruvate 10 micromol/L, glutamate 1 micromol/L, glycerol 10 micromol/L. The high frequency ICP, CPP, PRx, and PbtO_2_ signals were downsampled to minute-by-minute format and then combined in epochs corresponding to the times of the sequential microdialysis assays, allowing for the 17 minute transit from the microdialysis catheter membrane to the collection vial [10].

To mitigate any bias of a small proportion of extreme outliers when calculating summary statistics the data were Winsorized at the 0.00125 quantile, i.e. data that were either above or below the 99.875th and 0.125th quantile, respectively, were changed to be equal to the respective quantile threshold. This does not remove data (unlike trimming) but allows more robust calculation of parametric statistics.

### Statistical analysis

All statistical analysis was performed in R (v.3.4.3; www.r-project.org) using the packages: *nlme*, *rms*, *mgcv*, *mice*, *survival*, *euler*, *and ggplot2*. Ordinal proportional hazards regression was performed with the *lrm* function from the *rms* package. A substantial proportion of patients had at least one missing data point which would result in exclusion from cohort-level regression. Therefore, for these analyses the missing data were multiply imputed using a fully conditional chained equation model with the *mice* R package [11]. Continuous variables were imputed with predictive mean matching, and factors with logistic regression. Each of ten imputed datasets were then each entered into the regression model(s) and the results pooled to obtain overall estimates of coefficients and standard errors.

For analysis of the hourly data linear mixed-effects (LME) and generalised additive mixed models (GAMM) were used [12, 13]. The latter of these is an extension to the mixed modelling framework to include non-linear smooth functions of the independent variables being studied, represented in spline basis functions. This allows data-driven estimation of non-parametric non-linear relationships between variables. Furthermore, patient-level random effects and autoregressive error terms can also be included. GAMMs are therefore well served to analyse time-series/longitudinal data of differing duration from multiple individuals. The basis for smooth terms was a cubic regression spline with ten knots; random effects for each patient together with a continuous-time autoregressive term (CAR1) were included in the *gamm* function from the *mgcv* package. Restricted Maximum Likelihood (REML) was used in all *gamm* analysis. Comparison between temporal trends was examined with F-tests. To identify regions of non-linear trends where there was significant change in the dependent variable, the first derivative of the fitted GAMM model was determined by a finite difference method [14]. Where the 95%CI of the respective first derivative did not include zero the region of the curve was considered to represent a changing trend in the dependent variable. Where the first derivative did include zero the curve was considered to be ‘static’.

### Role of the funding source

The funders of this study had no role in design, data collection, analysis, or interpretation of the results.

## Results

### Demographics

Data for a total of 619 patients were collated for the study; baseline characteristics and outcomes are summarised in Table 1.

**Table 1:** Admission characteristics and outcomes of the patient cohort.

		Total N = 619
**Age**		*(median [IQR] years)*
		37 [24–52]
**Sex**		*(N [%])*
	Male	472 [76.3]
	Female	147 [23.7]
**Glasgow Coma Scale**		*(N [%])*
	3–8	387 [62.5]
	9–12	104 [16.8]
	13–15	86 [13.9]
	N.A.	42 [6.7]
**Pupils**		*(N [%])*
	Both reactive	417 [67.4]
	Unilateral unreactive	69 [11.1]
	Bilateral unreactive	21 [3.4]
	N.A.	112 [18.1]
**Marshall Computed Tomography Score**		*(N [%])*
	Class II	298 [48.1]
	Class III	92 [14.9]
	Class IV	21 [3.4]
	Class V	96 [15.5]
	Class VI	36 [5.8]
	N.A.	76 [12.3]
**Mechanism of Injury**		*(N [%])*
	Assault	53 [8.6]
	Fall	200 [32.3]
	Cyclist	37 [6.0]
	Pedestrian RTA	51 [8.2]
	Occupant RTA	260 [44.9]
	Other and N.A.	18 [2.9]
**Glasgow Outcome Score**		*(N [%])*
	1 (Death)	122 [19.7]
	2 (Vegetative State)	6 [1.0]
	3 (Severe Disability)	126 [20.4]
	4 (Moderate Disability)	191 [30.9]
	5 (Good Outcome)	137 [22.1]
	N.A.	37 [6.0]

IQR, interquartile range; N.A., not available.

GCS and pupillary reaction at initial presentation were not available from electronic records for 42 (6.7%) and 112 (18.7%) of patients, respectively. Outcome could not be determined in 37 (6.0%) of patients. The median time to the initiation of microdialysis monitoring was 26.9 [16.5–50.1] hours and was continued for a median IQR] of 4.8 [2.7–7.8] days. In total there were 56,307 microdialysis epochs included, with a median sampling interval of 1.02[1.00–1.40] hours. In addition to microdialysis, 461 (74.5%) patients had ABP, ICP, and PbtO_2_ data recorded in ICM+.

### Lactate/Pyruvate ratio and outcome

**Fig 1:**
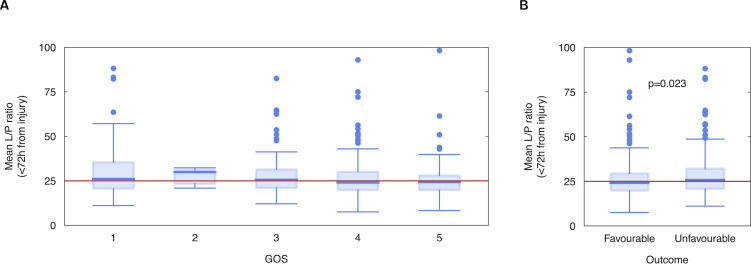
Boxplots of individual patients’ mean lactate-pyruvate ratio (LPR) over the first 72h following injury, by (A) Glasgow Outcome Score (GOS) and (B) dichotomised GOS.

Univariate comparison of individual mean LPR was not significantly different between unfavourable and favourable outcome groups when averaged over the first 24h from injury but was significantly different when averaged over the first 72h from injury (Fig 1 median [IQR]: 25.8 [11.0] vs. 24.2 [0.6], p = 0.023) and significantly higher proportion of time at LPR>25 (median [IQR]: 50.6% [90.0] vs 29.8% [79.0], p = 0.047). When averaged over the whole monitoring period there was again no significant difference between groups.

To examine the independent effect of LPR and other physiological parameters on outcome a proportional odds logistic regression of the five-point GOS outcome scale against age, presenting GCS, pupillary reaction, Marshall CT score, and <24h to <168h mean values of glucose, LPR, ICP, CPP, PRx, and PbtO_2_ was conducted. Tenfold multiple imputation of missing data using chained equations was performed and the proportional odds regression repeated and pooled; the results are shown in Table 2.

**Table 2:** Proportional odds regression against Glasgow Outcome Score (GOS) of baseline characteristics, microdialysis analytes, and monitoring parameters averaged over different intervals following injury.

	≤24h	≤72h	≤120h	≤168h	All
**Age**	- 0.032 (0.006)***	-0.033 (0.005)***	-0.031 (0.005)***	-0.031 (0.005)***	-0.031 (0.005)***
**GCS 9–12**	-0.221 (0.294)	-0.168 (0.275)	-0.163 (0.274)	-0.157 (0.280)	-0.171 (0.285)
**GCS ≤ 8**	-0.707 (0.246)**	-0.692 (0.227)**	-0.708 (0.229)**	-0.688 (0.236)**	-0.726 (0.238)**
**Pupils (Unilateral)**	-0.410 (0.271)	-0.461 (0.301)	-0.459 (0.290)	-0.454 (0.288)	-0.444 (0.282)
**Pupils (Bilateral)**	-1.728 (0.435)***	-1.791 (0.408)***	-1.712 (0.404)***	-1.661 (0.405)***	-1.623 (0.410)***
**Marshall III**	-0.314 (0.275)	-0.210 (0.254)	-0.260 (0.258)	-0.254 (0.262)	-0.248 (0.279)
**Marshall IV**	-0.638 (0.409)	-0.429 (0.460)	-0.404 (0.455)	-0.365 (0.456)	-0.339 (0.455)
**Marshall V**	0.029 (0.224)	-0.057 (0.214)	-0.088 (0.208)	-0.095 (0.210)	-0.117 (0.210)
**Marshall VI**	-1.015 (0.326)**	-0.893 (0.342)**	-0.814 (0.345)*	-0.876 (0.340)*	-0.854 (0.346)*
**Glucose**	-0.026 (0.074)	-0.082 (0.077)	-0.113 (0.071)	-0.147 (0.070)*	-0.186 (0.073)*
**LPR**	-0.006 (0.006)	-0.012 (0.006)*	-0.015 (0.007)⇤	-0.016 (0.007)*	-0.014 (0.007)*
**Glutamate**	-0.002 (0.003)	-0.004 (0.005)	-0.001 (0.005)	-0.002 (0.005)	-0.001 (0.005)
**Glycerol**	-0.000 (0.001)	-0.000 (0.001)	0.001 (0.001)	0.001 (0.001)	0.000 (0.001)
**ICP**	0.003 (0.028)	-0.061 (0.019)**	-0.057 (0.017)***	-0.064 (0.019)***	-0.078 (0.020)***
**CPP**	-0.001 (0.024)	-0.008 (0.012)	-0.002 (0.014)	0.000 (0.013)	0.009 (0.014)
**PRx**	-0.032 (0.574)	-1.668 (0.579)**	-1.921 (0.572)***	-2.215 (0.591)***	-2.602 (0.649)***
**PbtO2**	0.003 (0.010)	0.008 (0.008)	0.008 (0.008)	0.006 (0.007)	0.003 (0.008)
**C (AUROC)**	0.681	0.694	0.695	0.701	0.709

*** p < 0.001

** p < 0.01

* p < 0.05

Numbers are regression coefficients with standard errors in parentheses; statistical significance indicated by asterisk. Harrell’s C statistic, equivalent to the area under the receiver operating curve (AUROC) is also included for each model.

GCS, Glasgow Coma Scale; LPR, lactate/pyruvate Ratio; ICP, intracranial pressure; CPP, cerebral perfusion pressure; PRx, measure of autoregulation (see text); PbtO2, brain tissue oxygen tension.

At all timepoints age, low GCS (*<* 9), bilaterally unreactive pupils, and unevacuated mass lesion were significant predictors of outcome. Of the monitored variables, ICP, PRx, and LPR were significant predictors when averaged over 72h or longer. Cerebral glucose was also significantly associated with outcome when averaged over the first seven days from injury. As expected, the overall predictive power of the regression improved with time as evidenced by the increase in Harrell’s C-statistic, equivalent to area under the receiver operating curve for binary logistic regression.

### Temporal trends

**Fig 2:**
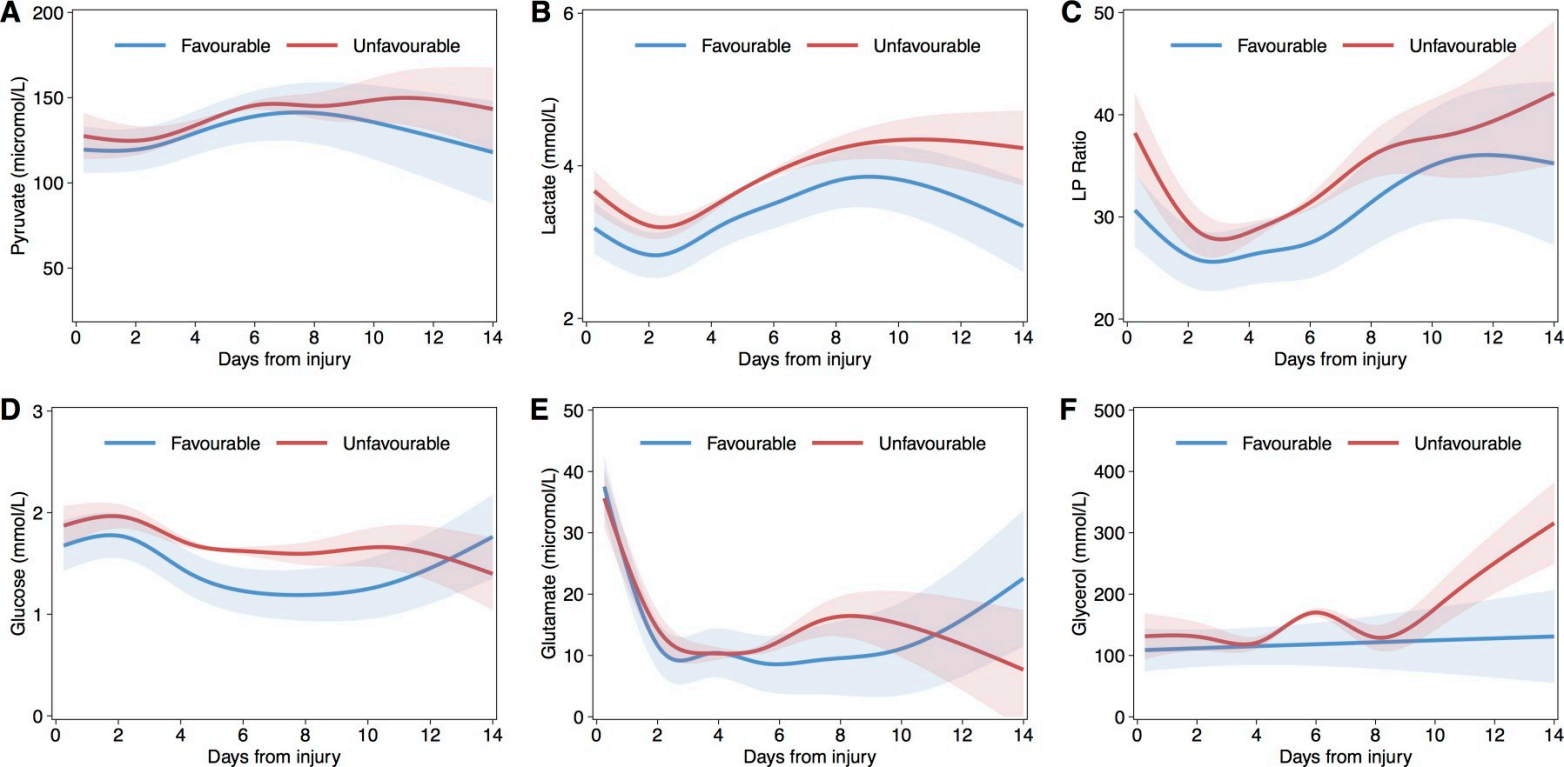
Fitted population mean time courses for microdialysis analytes using Generalised Additive Mixed Models (GAMM), by dichotomised glasgow outcome score group. Lactate (A), Pyruvate (B), Lactate-Pyruvate Ratio (LPR, C), Glucose (D), Glutamate (E), and Glycerol (F). Shaded areas indicated 95% confidence intervals.

**Fig 3:**
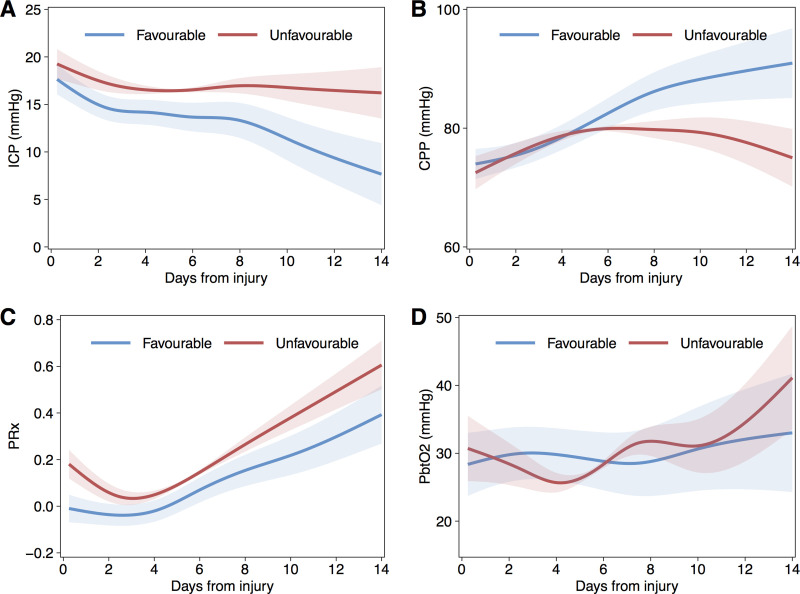
Fitted population mean time courses for intracranial pressure derived parameters and brain tissue oxygenation. Temporal course fitted with generalised additive mixed models (GAMM), by dichotomised Glasgow Outcome Score group. ICP (A), CPP (B), PRx (C), and PbtO_2_ (D), Shaded areas indicated 95% confidence intervals.

Generalized additive mixed models, including per-patient random effects and first-order autoregressive terms, were generated to fit data over first temporal profile of microdialysis analytes 14 days following injury. To ensure sufficient degrees of freedom to reflect the temporal trends without overfitting the smooth terms utilised a 7-knot cubic regression splines. Figs 2 and 3 shows the temporal profile of the monitored variables separated into favourable and unfavourable outcome categories.

LPR showed an initial decreasing trend over the first c.48h and then generally increased to day 14; these fluctuating changes over time were significant over for both favourable (p<0.001) and unfavourable (p<0.001) outcome groups but with significant differences in both overall mean intercept (3.40, SE[1.67], p = 0.0417) and trend between groups (p<0.001). Overall, LPR was on average higher in the unfavourable group until around day 10 following injury. Pyruvate exhibited a significant, and generally increasing, temporal trend but this did not differ significantly either in mean value or trend between outcome categories. In contrast, lactate was higher amongst the unfavourable outcome group throughout the period of monitoring. Of interest, cerebral glucose was significantly lower over days five to eleven following injury in the favourable outcome group. Both glutamate and glycerol were generally higher in the unfavourable outcome group but not significantly different. PRx demonstrated a similar temporal trend in both outcome groups but was significantly higher (reflecting more impaired autoregulation) in those with unfavourable outcome. ICP was significantly lower and decreased further in patients who went on to have a good outcome, and corresponded with the temporal trends of CPP. PbtO_2_ was similar between groups in the first week after injury but then was lower in the patients who ultimately had an unfavourable outcome.

### Relationship between LPR and glucose, CPP, PRx, and PbtO_2_

**Fig 4:**
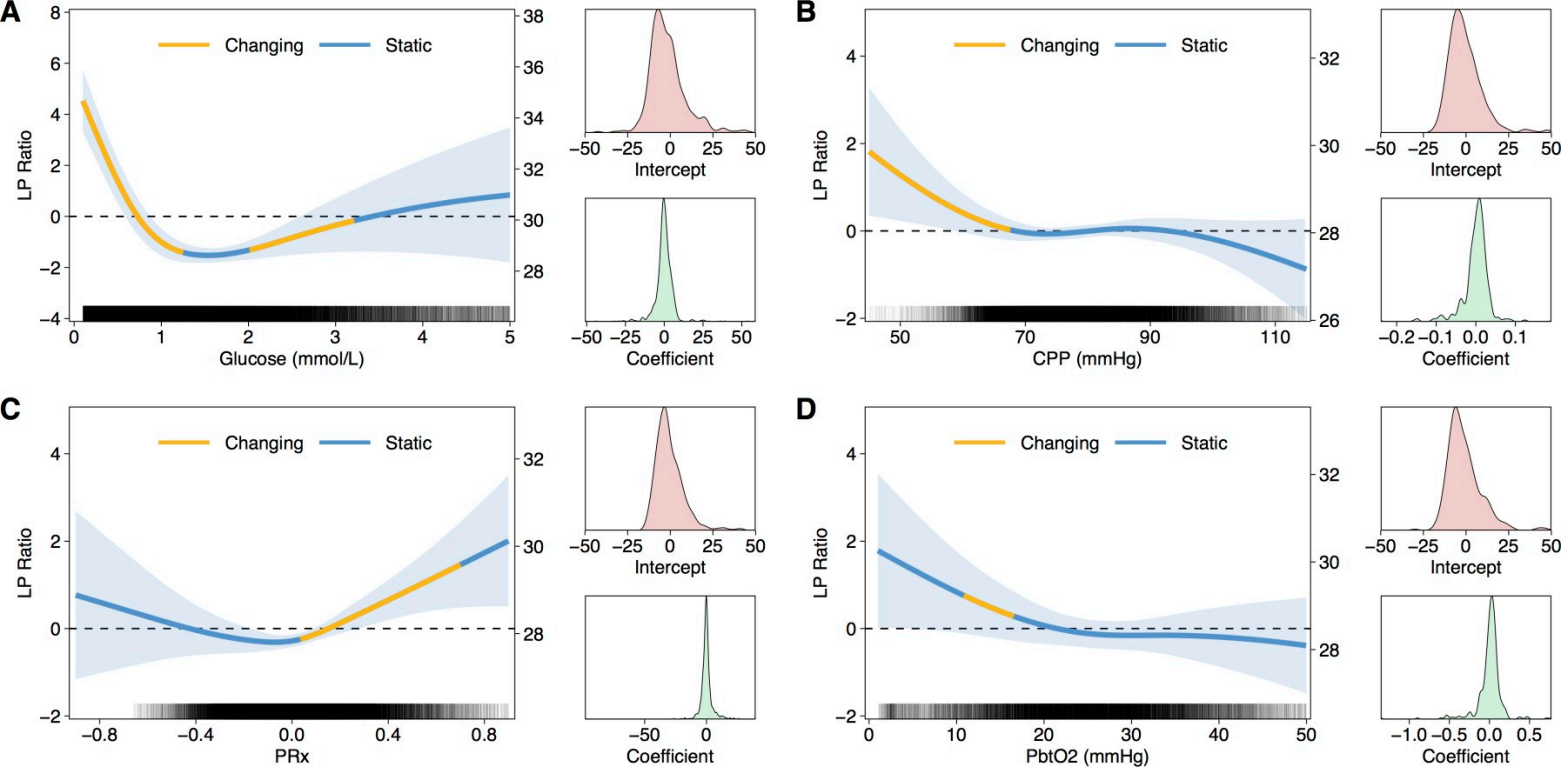
Relationship between lactate-pyruvate ratio (LPR) and other physiological parameters. In each panel a GAMM fit of glucose (A), CPP (B), PRx (C), and PbtO_2_ (D) against LPR is plotted. On the left axis LPR is scaled to zero mean without the intercept term; on the right axis the scale includes the overall intercept. Shaded region indicates 95%CI for the fit. The regions of each curve highlighted in yellow indicate non-zero first derivative i.e. where the is significant change. Alongside each plot is the distribution of random effects for intercept and slope/coefficient. Rug plot within in each panel indicates density of data.

To assess the associations between brain glucose, CPP, PRx, and PBtO_2_—as potentially modifiable parameters to improve cerebral metabolism—and LPR a further series of GAMMs were generated with LPR as the dependent variable. Random effects for intercept and slope, and continuous first-order autoregressive terms were again included. Importantly, all relationships were non-linear (Fig 4). Inspection of the GAMM models and derivatives suggested natural thresholds of the physiological parameters associated with rising LPR i.e. brain glucose below 1mmol/L, CPP below 60mmHg, PRx above 0.1, and PbtO_2_ below 18mmHg. Based on these thresholds a series of linear mixed models were used to compare mean LPR between dichotomised groups, including per-patient random effects and continuous autoregressive term. Consistent with the GAMM models, LPR was significantly higher when with glucose <1 mmol/L (mean LPR increase 1.33, SE 0.13, p<0.001) CPP <60mmHg (mean LPR increase 1.70, SE 0.43, p<0.0001), and PRx >0.1 (mean LPR increase 0.78, SE 0.14, p<0.0001). PbtO_2_ <18mmHg was associated with higher mean LPR (mean LPR increase 0.57, SE 0.37, p = 0.129) but was not statistically significant.

**Fig 5:**
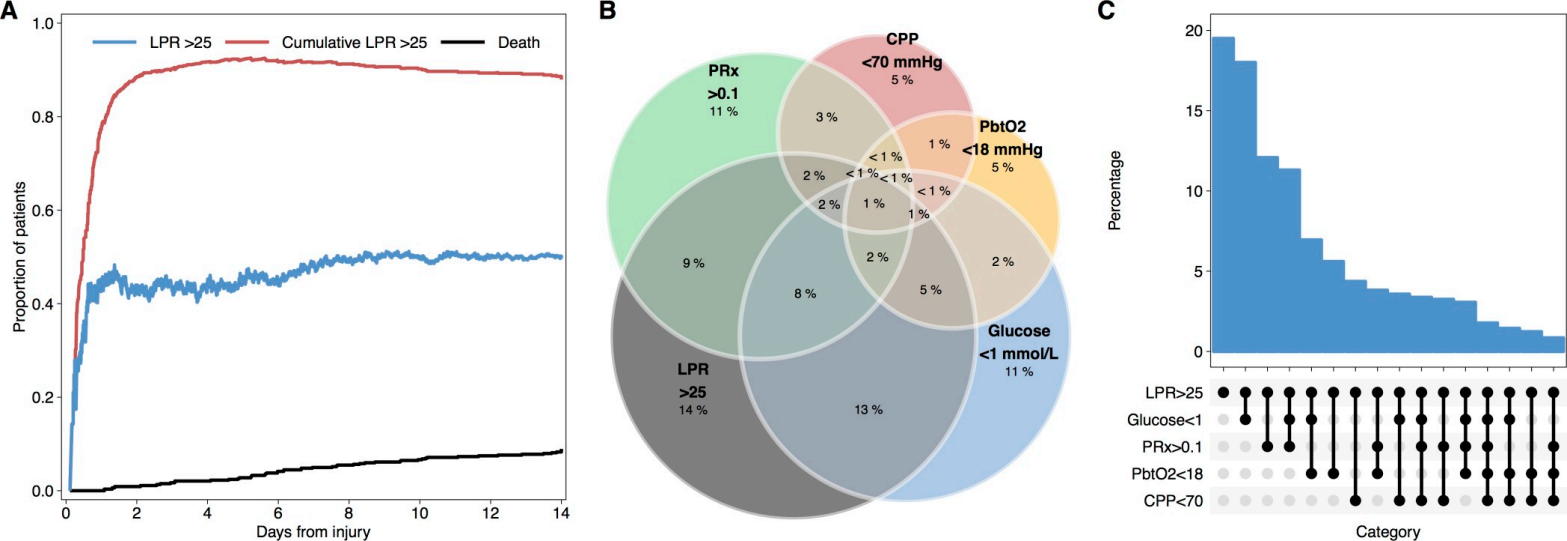
LPR elevation and associated physiological derangements. Survival based curves (A) indicating likelihood of LPR >25 and the cumulative incidence of at least one epoch of LPR>25. Euler plot (B) showing prevalence of abnormalities in brain glucose, CPP, PRx, and PbtO_2_ (defined by the thresholds described in the text) during episodes of elevated LPR. Alternative representation as ‘Upset’ plot (C) showing differing frequency of permutations of physiological abnormalities (as indicated in the panel beneath the x-axis) associated with elevated LPR.

Overall, 48.9% of microdialysis samples where pyruvate and lactate were assayed demonstrated LPR >25. The vast majority of patients had at least one epoch with LPR >25 during their monitoring period; after the first 24h from injury approximately half of patients at any one time exhibited LPR >25 (Fig 5A). Examining only monitoring periods with LPR, glucose, CPP, PRx, and PbtO_2_ data available, when LPR >25, at least one of the physiological parameters was outside of the thresholds defined above on 80.5% of occasions (Fig 5B and 5C).

## Discussion

### Cerebral metabolism and outcome

This study of combined microdialysis and neuromonitoring data in 619 TBI patients represents the largest series in the literature and builds on the evidence from previous studies, including the findings from the initial 223 patients in the cohort [8]. Cerebral LPR averaged per patient over the first three, five, or seven days following injury was found to be independently associated with neurological condition at six months following injury, measured ordinally on the GOS scale. This affirms findings in previous studies [7]. The lack of association between LPR and outcome when averaged over the first 24 hours or the whole period of monitoring likely reflects the relative lack of data in the early period following injury and the confounding factors and selection bias in patients who were monitored beyond seven days. It should be emphasised that the modelling presented here is not intended to motivate adoption of LPR (or any other microdialysis-derived parameter) as a specific or precise prognostic marker in the clinical management of individual TBI patients. Rather, the rationale for examining the association with outcome is to test the broad face-validity of the presumption that derangements of cerebral metabolism are related to outcome after TBI (and brain injury in general) and the corollary that therapy directed at normalising metabolism may have potential benefit.

Consistent with the regression analysis, the mean temporal course of LPR was significantly different over the first several days from injury between dichotomised outcome groups. Both groups exhibited a decrease in LPR over the initial 48 hours following injury, possibly consistent with recovery from any acute derangements of ICP and cerebral blood flow together with physiological stabilisation in the intensive care unit [15]. Thereafter LPR increased, with a more pronounced and variable pattern in poor outcome patients. At a mean level, the secondary increase in LPR was due primarily to increasing lactate, whereas the trend for pyruvate was relatively flat.

### Metabolic dysfunction following TBI

The canonical model of cellular respiration commences with glucose entering the cytosol, proceeding along the series of enzymes of the glycolytic pathway to pyruvate (with net gain of two ATP molecules), which in turn enters the mitochondrion to feed the TCA cycle and the electron transport chain, producing a further 34 molecules of ATP. When oxygen is insufficient, pyruvate is converted to lactate to regenerate NAD and allow anaerobic glycolysis to continue. In the CNS there is evidence that glia and neurons are metabolically coupled in the so-called ‘Astrocyte-Neuron Lactate Shuttle’ (ANLS) [16–18]. This model posits that neurons do not generally utilise glucose directly but predominantly generate pyruvate from lactate that is produced by glycolysis in astrocytes. During periods of greater neuronal activity and metabolic demand synaptic glutamate release increases, which is in turn taken up into surrounding astrocytes and upregulates glycolysis to generate further lactate that diffuses to neurons for utilisation in oxidative metabolism. Several compelling lines of evidence support the ANLS in animal models and humans but irrespective of the more complex arrangements, net brain metabolism remains predominantly oxidative in normal circumstances. In animals in-vivo, experimental evidence exists, in accord with the ANLS, for oxidative metabolism of lactate in the brain, in rats [19, 20], and for a lactate gradient from astrocytes to neurons, in mice [21]. Lactate, like many other brain metabolites, is in a state of flux, being both produced and removed. One removal mechanism is via TCA cycle metabolism [22]. Also it can be exported into the vasculature, evidenced by arterio-jugular venous difference (AJVD) measurements [23]. A third removal route is via the so-called glymphatic system–paravascular channels in the brain, which facilitate clearance of waste products, including lactate, from the brain interstitial fluid into the CSF and thence into lymph vessels [24]. This glymphatic clearance is enhanced during sleep or anaesthesia, compared to wakefulness [24]. Interesting questions thus arise of whether TBI damage to the glymphatics contributes to the rise in brain lactate, and whether glymphatic function could be optimised by adjustment of anaesthesia/sedation to regulate the brain LPR.

As evidenced in the present study, frank cerebral ischaemia—i.e. low cerebral perfusion pressure and/or tissue hypoxia—is uncommon with modern critical care management of arterial blood pressure, intracranial pressure, and ventilation. Nonetheless, accumulated evidence suggests a number of other forms of metabolic dysfunction commonly manifest after TBI, and different types may coexist dynamically or be regionally localised within an individual patient [5, 25, 26]. Even if cerebral blood flow is adequate, substrate delivery may be impaired by local diffusion barriers that result from cell necrosis and matrix breakdown. Aerobic hyperglycolysis, wherein anaerobic glycolysis is upregulated despite sufficient oxygen availability, has been demonstrated to be a common early phenomenon in both preclinical models and human TBI patients [27]. Overlapping to some extent is the concept of ‘mitochondrial dysfunction’, a situation where oxidative respiration cannot proceed effectively due to deficiencies in the TCA cycle or electron transport chain, with an obligate increase in the rate of glycolysis to maintain ATP production.

### Elevated LPR and other physiological parameters

A key achievement of this study is the demonstration of non-linear associations between LPR and brain glucose, CPP, PRx, and PbtO_2_. The flexibility and data-driven approach of GAMM modelling is a powerful method of estimating smooth non-linear relationships in data from multiple subjects while accounting for strong autocorrelations inherent in physiological time series. Furthermore, interrogating the derivatives of the fitted curves allows inference of natural threshold in the inter-relationships that can potentially inform treatment plans.

In the present data, glucose <1mmol/L, CPP <70mmHg, PRx >0.1, and PbtO_2_ <18mmHg were present in four fifths of periods when LPR was elevated >25. This suggests that targeted intervention to manipulate and correct these parameters may have the potential to improve cerebral chemistry. For example, the results implicate glucose concentration as a key determinant of cerebral metabolic state. The immediate question that follows is whether supplementing cerebral glucose can improve LPR and have a beneficial effect on patient outcome. Target ranges for plasma glucose in the neurointensive care unit are a subject of much debate and several studies have shown that tight control with continuous insulin infusions, as used in general intensive care, have been associated with reduced cerebral glucose and elevated LPR in TBI patients when compared with more permissive glycaemic control [28–30]. However, early hyperglycaemia in TBI patients is associated with poor outcome [31], and both intravenous and direct intracerebral glucose supplementation has not shown consistent effects on metabolism [32–34].

In contrast, the relationship between LPR and PbtO_**2**_ was found to be much less robust than CPP and PRx. These differences in findings are subject to the limitations of many individual variations, and that LPR, glucose and PbtO_**2**_ are focal measures within a small ROI of the brain, whereas CPP and PRx are effectively global measures relating to the brain vasculature. The weakness of the relationship between LPR and PbtO_**2**_ concords with the growing evidence that LPR>25 is not only a marker of hypoxia / ischaemia (which are nowadays uncommon, with modern neurocritical care), but LPR can become elevated even when PbtO_**2**_ levels are regarded as “adequate”–suggesting mitochondrial dysfunction. This concurs with our recent study [35] wherein application of a tiered protocol enabled the identification of neurometabolic states. Within that study, in those patients with LPR>25, mitochondrial dysfunction and neuroglycopaenia were most frequent. In the present data, adopting the thresholds from the GAMM analysis, approximately 20% of periods of elevated LPR were not associated with derangements in other parameters and it may be this subset that represents mitochondrial dysfunction. The relative scarcity of intracranial hypertension, delivery failure and tissue hypoxia may reflect the efficiency of modern neurointensive care in identifying and treating these physiological derangements, which are monitored continuously in real-time, allowing rapid targeted corrective response by intensivists–whereas LPR and glucose in brain microdialysates are measured hourly, and blood glucose less frequently.

### Limitations

This study has a number of limitations inherent to observational investigations. Missing data was addressed with multiple imputation but this is imperfect and possibility of bias remains. Although the general management protocol for TBI patients at our institution is standardised, the specific interventions and their timing for each patient was not recorded and therefore potential confounding effects on the data are not quantifiable. However, this is in part mitigated by the large number of patients included. It is important to state that the differences in time course of microdialysis and pressure-related variables between favourable and unfavourable outcome groups could both reflect a contrasting spectrum of severity of injury amongst patients, but alternatively may also be a consequence of varying patient responses to similar degrees of injury. Despite the strong associations between variables observed, extrapolation to the effects of manipulating, for example, glucose concentrations, are only hypothetical and speculative; further interventional studies are required. Further work is needed to examine if the nonlinear functional form of the relationships between LPR and other physiological parameters remains consistent over time from injury, whether they differ in, for example, peri-contusional brain compared with radiologically normal tissue, and if affected by age, comorbidities, or other patient factors. These are key questions to address in taking forward the present findings to inform clinical treatment of patients.

### Research in context—evidence before this study

Baseline presenting features including age, Glasgow Coma Scale, and pupillary reactivity are established predictors of outcome following TBI. Physiological parameters monitored during the course of intensive care management, in particular intracranial pressure (ICP), have also been shown to be strongly related to outcome and the current standard of care is invasive ICP monitoring with treatment principally directed at preventing raised ICP and correspondingly reduced cerebral perfusion pressure (CPP). Additional monitors and indices, including brain tissue oxygenation (PbtO_2_), cerebral metabolism, and autoregulation (PRx) have been shown in a number of observational studies to have independent association with neurological outcome but the therapeutic strategies to optimise these parameters have not been well defined.

### Added value of this study

This study represents the largest cohort of TBI patients studied with microdialysis to date. It confirms the independent association of brain metabolic derangements, specifically raised lactate/pyruvate ratio (LPR), with survival and neurological outcome and therefore that therapy directed at normalising metabolism has the potential to improve outcomes. The non-linear relationships identified between LPR and CPP, PbtO_2_, PRx, and brain glucose directly suggest therapeutic goals that could be adopted to improve cerebral metabolism.

### Implications of all the available evidence

Optimising the prevention of secondary brain injury and improving outcomes following TBI is dependent on monitoring key physiological parameters and having available effective therapeutic interventions to correct them. ICP remains the primary target of modern intensive care TBI management but the present study suggests that there could be significant additional gains achieved by addressing cerebral metabolic derangement. Prospective interventional studies are required to establish the efficacy of such strategies.

## Conclusions

Cerebral metabolic dysfunction, as evidenced by raised LPR, is associated with patients’ neurological outcome following TBI. Low CPP and PbtO_2_ should be avoided as per current guidelines. Targeting CPP to minimise PRx (i.e. optimise autoregulation) may be beneficial in ameliorating metabolic dysfunction. Glucose is strongly related to both lactate and pyruvate concentrations and LPR; the benefit of supplementing glucose in patients with raised LPR warrants further research.
